# Early-life inequalities and biological ageing: a multisystem Biological Health Score approach in *U* *nderstanding* *S* *ociety*

**DOI:** 10.1136/jech-2018-212010

**Published:** 2019-04-03

**Authors:** Maryam Karimi, Raphaële Castagné, Cyrille Delpierre, Gaëlle Albertus, Eloïse Berger, Paolo Vineis, Meena Kumari, Michelle Kelly-Irving, Marc Chadeau-Hyam

**Affiliations:** 1 Department of Epidemiology and Biostatistics, School of Public Health, Imperial College London, London, UK; 2 MRC-PHE Centre for Environment and Health, Imperial College London, London, UK; 3 UMR1027, Université de Toulouse, UPS, Inserm, Toulouse, France; 4 Italian Institute for Genomic Medicine IIGM, Torino, Italy; 5 Institute for Social and Economic Research, University of Essex, Colchester, UK

**Keywords:** biomarkers, biological ageing, social epidemiology

## Abstract

Social position is known to play a role in the quality of ageing, notably through the stimulation/dysregulation of key physiological systems in response to external stresses. Using data from one wave of *Understanding Society* including 9088 participants, we defined, as an extension of the allostatic load, a synthetic Biological Health Score (BHS) capturing the wear-and-tear of four physiological systems (endocrine, inflammatory, cardiovascular and metabolic systems) and two organs (liver and kidney). We used 16 established blood-derived biomarkers of these systems to calculate the BHS and explored the relative contribution of socioeconomic position to the BHS and its main components across age groups. We identified a systematic decreasing education-related gradient of the BHS (p<0.001) leading to lower biological risk in participants with longer education. Education-related differences in the BHS were detected early in life, and were not attributable to lifestyle and behavioural factors. We found a consistent contribution of the inflammatory and metabolic systems to the overall score throughout from early adulthood onwards, while the contribution of the other four systems seems to vary across age groups and gender. Our findings highlight the social-to-biological processes ultimately leading to health inequalities, and suggest that such disparities can already be detected in the 20–40 years old age group and cannot be fully explained by lifestyle and behavioural factors. This may define early adulthood social condition as a precursor to accelerated biological ageing and as an important target for public health policies.

## Introduction

The basic requirements for human life rely on the adequate function of biological systems, allowing for cognitive and physical performance, and overall well-being. There is increasing evidence that physiological functioning is socially patterned,^1–3^ suggesting that living in disadvantaged social conditions leads to physiological adaptation that may have a cost in terms of physiological dysregulation developing over time. This is likely to contribute to unhealthy ageing, notably through subsequent inflation of the individual risk of chronic diseases.

Questions around how environmental factors lead to biological alterations over time can now be investigated on a wider scale, and in a variety of contexts and disciplines.^4^ One of the main issues is how to measure socioeconomic-related physiological changes at different life stages, that is, in relation to biological wear-and-tear, or to biological ageing, rather than in relation to specific pathologies. The relationships between molecular markers, physiological systems and overall physical and cognitive performances are under increased scrutiny by researchers attempting to elucidate the mechanisms involved in predisease and even ‘normal’ physiological contexts.^5–7^ In this framework, ageing is modelled as a progressive decline of the integrity and/or level of functioning across multiple organ systems.^5^ Due to the complexity of the ageing process and to the pleiotropic contribution of several systems to the quality of ageing, the use of multisystem scores has been proposed and appears to provide an efficient and interpretable alternative to the investigation of (sets of) prioritised biomarkers.^8^

The allostatic load (AL) as originally defined is a composite score measuring the lifelong physiological wear-and-tear mainly related to stress response. Developments beyond the concept of AL^9^ have demonstrated that socioeconomic adversity, particularly in early life, leads to a higher load (ie, higher lifelong stimulation of several key physiological systems), which has been related to increased risk of health outcomes such as mortality, morbidity and unhealthy ageing.^10–12^

Building on the concept of the AL, we hypothesise here that socioeconomic differences in biological health occur throughout the age range of the population, hence suggesting that socially stratified physiological differences could be detected from early life and could persist over the life course, and could therefore help in better understanding the ageing processes.^13^ Using data from *Understanding Society*, we adopt the same methodology as that developed for the AL and include liver and kidney functions which were available in the study and represent non-primarily stress-related systems involved in human physiological wear-and-tear.^8 14^ We use our extended definition of the AL and first explore the evolution of the score across age groups. We subsequently assess if there is a systematic Biological Health Score (BHS) gradient related to education, and quantify, among the six systems considered in our score, those mostly contributing to the BHS and to its age and education-related variation. Finally, we investigate whether observed BHS differentials are driven by established lifestyle and behavioural factors.

## Materials and methods

### Study population

Beginning in 2009–2010, *Understanding Society*, or the UK Household Longitudinal Study, is an annual longitudinal survey of more than 40 000 UK households. It consists of a general population sample (GPS), a stratified clustered random sample of households representative of the UK population, as well as a smaller component from the pre-existing British Household Panel Survey. Annual interviews collecting sociodemographic information are conducted throughout each year. In this study we used a subset of the GPS sample who participated in a single nurse visit when physical measures and blood samples were collected. This took place in the participant’s home 5 months after the annual wave 2 interview in 2010–2012. Respondents were eligible for a nurse visit if they had taken part in that wave’s main interview in English; were aged 16+; lived in England, Wales or Scotland; and were not pregnant. Of 35 875 participants meeting these requirements, 15 591 adults took part in wave 2 of the nurse health assessment, and blood samples were collected from 10 175 participants.^15^

### Socioeconomic position

Educational level captures the length of formal education and characterises the qualification, and can therefore be viewed as an indicator of socioeconomic position that jointly relates to social class, social status and material circumstances. In the present study we used participants’ or their parent’s educational level as a six-level categorical variable. We regrouped these levels into three groups: low (no qualification), intermediate (GSCE (General Certificate of secondary Education) and so on, other qualification, A level and so on) and high (degree and higher degree) for individual education. For parental educational level the new groups were low education (did not go to school at all, left school with no qualifications or certificates), intermediate (left school with some qualifications or certificates, other) and high (gained post school qualifications or certificates, a university degree or higher degree). As a measure of Socio Economic Position (SEP), individuals aged younger than 25 years were attributed the highest achieved parental or individual educational level, and participants aged 25 or older are attributed their own educational level.

### Biomarkers

Based on a systems approach reconciling the literature on AL, biological ageing and the human disease network,^15–18^ we selected a panel of 15 (women) or 16 (men) biomarkers measured in the blood samples collected at the nurse health assessment. These related to four systems: endocrine, cardiovascular, metabolic and inflammatory; and to the function of two organs: liver and kidney (see box 1).

Box 1 **Definition and calculation of the Biological Health Score (BHS) (A) and system-specific scores (B)**

A. Definition of the BHS.

- The BHS is calculated using the distribution of the (n=16) biomarkers included in the study and targeting four physiological systems (as included in the allostatic load) and two organs:

- *Endocrine system* (n=1/2 biomarkers in women and men, respectively): dehydroepiandrosterone sulfate (DHEA-S) and testosterone (in men only).
- *Metabolic system* (n=4): glycated haemoglobin, high-density lipoprotein cholesterol (HDL), total cholesterol and triglycerides.
- *Cardiovascular system* (n=3): systolic and diastolic blood pressure, and pulses.
- *Inflammatory/Immune system* (n=3): C reactive protein, fibrinogen and insulin-like growth factor 1 (IGF-1).
- *Liver function* (n=3): alanine transaminase, aspartate transaminase and gamma glutamyltransferase.
- *Kidney function* (n=1): creatinine.

- For a given individual i, the BHS is calculated as the sum (across all 16 biomarkers) of binary variables indicating if that person belongs to the ‘at-risk’ quartiles of each biomarkers.

$BHS^{i}=\sum_{k=1}^{16}I_{k}^{i},where$

krepresents the biomarker, and $I_{k}^{i}$ the binary variable informing if levels of biomarker k measured in individual i are in the at-risk quartile.

- The ‘at-risk’ quartile is defined as the lowest quartile for DHEA-S, testosterone, HDL and IGF-1, and the highest quartile for all remaining 12 biomarkers.
- Quartiles are calculated for both genders and each age group separately.
- The BHS measures the cumulated biological risk (across systems and/or biomarkers) a participant of a given age and gender is subjected to.

B. Definition of the system-specific subscores.

- To investigate the relative contribution of each system system-specific subscores are calculated as the sum of the same binary variables, but restricted to the system they measure. To ensure comparability across system-specific subscores, these are standardised by the number of biomarkers measuring that system. Specifically, for individual i:

${BHS}_{s}^{i}=\frac{\sum_{k=1}^{n_{s}}I_{k}^{i}}{n_{s}}\text{, where}$

${BHS}_{s}^{i}$is the system-specific subscore for system s, and $n_{s}$ is the number of biomarkers in system s.

### Definition of the BHS

We defined biological risk by using the sample distribution of each biomarker based on the method used for calculating the AL,^19^ stratified by age group and gender (see box 1). We included in the BHS all systems originally included in the AL, and additionally included kidney and liver functions. An individual is considered to be ‘at risk’ for a given biomarker if his/her measured value of that biomarker is in the extreme quartile of the empirical distribution of that biomarker in the age group and gender the individual belongs to. If an individual is defined as being ‘at risk’ for a biomarker, he/she is attributed a subscore of 1 for that particular biomarker and 0 otherwise. The individual BHS is derived by summing biomarker-specific scores across all biomarkers. The overall BHS then reflects the level of biological risk per age group and gender (see box 1-A).

In order to quantitatively compare the contribution of each system to the overall score, we also calculated a per-system score by summing biomarker-specific scores across all biomarkers within each system, and scaled the system-specific subscore by the number of biomarkers involved in each system (see box 1-B).

### Covariates

Marital status is a categorical variable in four groups: single, living as couple or married, separated or divorce, and widowed. Overcrowding was defined as the number people per room in the household (categorical, binary: <1.5 person per room or ≥1.5 person per room).^20^ Comorbidities originally included 17 diseases and were categorised here into three groups: none, one, and two or more comorbidities. Medicines and treatments were originally coded as binary variables describing if the participant reported the use of a treatment for 10 different types of pathologies, and were recoded into none, one, and two or more reported treatments. Smoking, physical activity and alcohol consumption were used as originally coded in the study. For smoking, a binary variable was used indicating if the participant had ever smoked. Physical activity was a binary variable indicating if the participant practised one or more sports. Alcohol consumption was grouped into four categories (non-drinker, less than two times in the last year; social drinker, once or twice a month; moderate drinker, once or twice a week; and daily drinker, 3 days a week or more). Body mass index (BMI, kg/m^2^) was defined using the WHO five groups: <18.5, (18.5, 25), (25, 30), (30, 40) and ≥40.

### Statistical analysis

Age grouping was defined to ensure that all age groups included a sufficient and comparable (online supplementary table 1) number of participants, and four categories were considered: 20–40, 41–52, 53–64 and 65–79 years old. Individuals with all missing biomarkers were excluded from the analyses. If the value of a given biomarker was missing, a null subscore was allocated to the individual for that specific biomarker.

10.1136/jech-2018-212010.supp1 Supplementary data

In order (1) to capture the reported differential social health gradients in men and women and (2) to investigate age ranges at which these could be detected, we first investigated the values of the BHS (and of each system-specific subscores) by age group and gender, and education group separately. Specifically, we calculated the mean BHS for each age group a, gender g, and obtained three estimated mean BHS values: ${\hat{BHS}}_{a,g,L}$, ${\hat{BHS}}_{a,g,I}$ and ${\hat{BHS}}_{a,g,H}$, for low, intermediate and high SEP groups, respectively. These three values were compared in each age and gender category using a Student’s t-test and using the value in the high SEP group (${\hat{BHS}}_{a,g,H}$) as a reference. We also tested if these three values for each age group and gender were supportive of a trend across SEP groups using a non-parametric Kruskal-Wallis rank test. These analyses were also performed on each system-specific subscore.

In order to evaluate the effect of SEP on the changes in mean BHS, taking into account possible confounders, we used the following model, for each age group and gender separately (ie, pooling data across SEP groups):

$$BHS_{i,a,g}=\alpha +\beta_{a,g}^{SEP_{i}}\times SEP_{i}+\beta_{a,g}^{FE_{i}}\times FE_{i}+\varepsilon_{i},where$$

${FE}_{i}$combines the observed values of the set of potential confounders for individual i, and ${SEP}_{i}$ is the SEP category individual i belongs to. In that setting, $\beta_{a,g}^{Low}$ and $\beta_{a,g}^{Intermediate}$, the estimates of the adjusted effect of SEP on the BHS for the low and intermediate SEP categories, respectively, can be interpreted as the average difference in BHS in the low and intermediate SEP groups compared with what is observed in the high SEP group, independently of the adjustment variables.

In a benchmark model (model 1), we did not include any adjustment variable in FE, and sequentially adjusted the model for the following covariates:

- Model 2=model 1 + marital status and overcrowding.
- Model 3=model 2 + comorbidities.
- Model 4=model 3 + medicines and treatments.
- Model 5=model 4 + smoking, physical activity and alcohol consumption.
- Model 6=model 5 + BMI.

We considered throughout a nominal significance level of 0.05. Statistical analyses were performed using R V.3.4.0 in the RStudio environment.

## Results

### Study population and BHS distribution

Of the original 10 175 eligible participants, 281 did not have full information from their interview and/or nurse health assessment. Of the remaining 9894 participants, 10 were excluded as not being in year 2 of primary sampling unit. We also restricted the age range of our study population and excluded (n=311) participants aged younger than 20 and (n=457) older than 79. Of the remaining 9116 participants, we excluded 1 due to outlying biomarkers measurement (in the last quartiles for 15 of 16 biomarkers), 20 due to all 15 of 16 biomarkers missing, 6 due to missing information on education and 1 due to aberrant BMI value. This resulted in a total of 9088 (3992 men and 5096 women) participants being included in our study, and their main characteristics are presented in online supplementary table 1. Of the (n=315) participants aged 20–24, 163 were allocated parental educational level and 152 their own. Younger age groups had a smaller proportion of individuals in the low education group compared with the older age groups. As expected, the number of comorbidities and treatments is higher in older age groups in both men and women. The proportion of individuals who have ever smoked is almost the same in different age groups for women, but is higher for older men compared with young men. Online supplementary table 2 presents the summary statistics and the percentage of missing values for each biomarker by age group and gender. A majority of participants included in the study did not have any missing biomarkers value (>64%); we excluded 20 participants with all 15 of 16 biomarkers missing, and only 295 (149 men and 146 women) had 4 or more missing (and subsequently imputed) biomarker measurements (online supplementary table 3).

10.1136/jech-2018-212010.supp2 Supplementary data

10.1136/jech-2018-212010.supp3 Supplementary data

The BHS distribution calculated across all age groups shows clear differences for each covariate (p<0.0001) except household overcrowding (p=0.444) (table 1). This indicates higher BHS values in men, in smokers, in participants who were separated, divorced or widowed, and in participants with lower education, comorbidities and medical treatments, higher BMI, and low physical activity.

**Table 1:** Summary statistics for the Biological Health Score (BHS) calculated by age group

	Age groups				
	20–40	41–52	53–64	65–79	Total sample
	(n=2276)	(n=2380)	(n=2298)	(n=2134)	(N=9088)
	Mean (SD)	Mean (SD)	Mean (SD)	Mean (SD)	Mean (SD)
Gender					
Male	3.914 (2.731)	4.016 (2.493)	4.033 (2.427)	3.955 (2.212)	3.981 (2.466)
Female	3.707 (2.447)	3.735 (2.609)	3.697 (2.363)	3.828 (2.212)	3.738 (2.424)
P value	0.237	0.002	0.001	0.202	<0.0001
Education					
Low	4.988 (2.926)	4.517 (2.651)	4.527 (2.318)	4.184 (2.340)	4.386 (2.422)
Intermediate	4.073 (2.639)	4.050 (2.504)	3.890 (2.405)	3.854 (2.147)	3.971 (2.438)
High	3.482 (2.437)	3.503 (2.585)	3.464 (2.350)	3.642 (2.150)	3.510 (2.416)
P value	<0.0001	<0.0001	<0.0001	0.0005	<0.0001
Marital status					
Single	3.455 (2.554)	3.936 (2.640)	3.979 (2.540)	3.602 (2.083)	3.638 (2.538)
Living as couple or married	3.935 (2.558)	3.791 (2.534)	3.758 (2.371)	3.834 (2.213)	3.826 (2.429)
Separated or divorced	3.775 (2.729)	4.154 (2.667)	4.089 (2.411)	4.284 (2.197)	4.126 (2.485)
Widowed	2.750 (2.061)	3.250 (2.468)	4.308 (2.489)	3.955 (2.236)	3.994 (2.308)
* *P value	0.0003	0.082	0.027	0.025	<0.0001
Household overcrowding					
No	3.754 (2.553)	3.808 (2.569)	3.870 (2.388)	3.902 (2.223)	3.835 (2.436)
Yes	3.947 (2.640)	4.148 (2.524)	3.564 (2.519)	3.716 (2.054)	3.916 (2.520)
P value	0.190	0.010	0.055	0.460	0.444
Comorbidities					
None	3.535 (2.444)	3.352 (2.382)	3.290 (2.237)	3.406 (2.107)	3.411 (2.346)
One	4.072 (2.659)	4.198 (2.565)	3.804 (2.360)	3.730 (2.134)	3.938 (2.428)
Two or more	5.318 (2.818)	5.032 (2.752)	4.550 (2.440)	4.259 (2.268)	4.564 (2.478)
P value	<0.0001	<0.0001	<0.0001	<0.0001	<0.0001
Number of treatments					
None	3.539 (2.505)	3.449 (2.432)	3.326 (2.279)	3.489 (2.146)	3.460 (2.404)
One	3.899 (2.520)	3.784 (2.438)	3.772 (2.271)	3.529 (2.017)	3.747 (2.321)
Two or more	5.069 (2.685)	4.970 (2.721)	4.432 (2.482)	4.180 (2.278)	4.476 (2.482)
P value	<0.0001	<0.0001	<0.0001	<0.0001	<0.0001
Smoking					
Never	3.613 (2.541)	3.623 (2.493)	3.733 (2.404)	3.711 (2.181)	3.666 (2.421)
Yes	3.917 (2.584)	4.030 (2.607)	3.918 (2.392)	3.990 (2.224)	3.963 (2.455)
P value	0.004	0.0002	0.054	0.007	<0.0001
Sports activity					
At least one sport	3.762 (2.573)	3.690 (2.510)	3.671 (2.414)	3.771 (2.188)	3.722 (2.443)
None	3.889 (2.562)	4.217 (2.651)	4.140 (2.343)	4.051 (2.237)	4.086 (2.432)
P value	0.239	<0.0001	<0.0001	0.0057	<0.0001
Alcohol consumption					
Non-drinker	4.310 (2.752)	4.794 (2.851)	4.331 (2.638)	3.984 (2.119)	4.338 (2.596)
Social drinker	3.900 (2.533)	4.297 (2.679)	4.157 (2.379)	3.977 (2.250)	4.073 (2.477)
Moderate drinker	3.668 (2.636)	3.518 (2.407)	3.783 (2.353)	3.864 (2.149)	3.694 (2.410)
Daily drinker	3.687 (2.499)	3.480 (2.431)	3.496 (2.310)	3.707 (2.245)	3.580 (2.362)
P value	0.006	<0.0001	<0.0001	0.068	<0.0001
Body mass index (kg/m^2^)					
Under 18.5	2.200 (1.728)	2.214 (1.477)	2.692 (1.652)	3.714 (1.267)	2.543 (1.674)
18.5 and below 25	2.636 (1.973)	2.446 (1.813)	2.643 (2.007)	2.799 (1.783)	2.619 (1.908)
25 and below 30	3.737 (2.277)	3.587 (2.266)	3.589 (2.138)	3.818 (2.031)	3.680 (2.179)
30 and below 40	5.633 (2.600)	5.173 (2.647)	4.971 (2.369)	4.641 (2.355)	5.072 (2.513)
Above 40	6.912 (2.361)	6.505 (2.452)	5.818 (2.383)	5.773 (2.400)	6.301 (2.434)
P value	<0.0001	<0.0001	<0.0001	<0.0001	<0.0001

Results are presented for each gender and each class of the categorical covariates. Differences in BHS across covariate categories (1) within each age group and (2) across all age groups (last column) were investigated using a Kruskal-Wallis rank test and the corresponding p values are reported.

**Figure 1:**
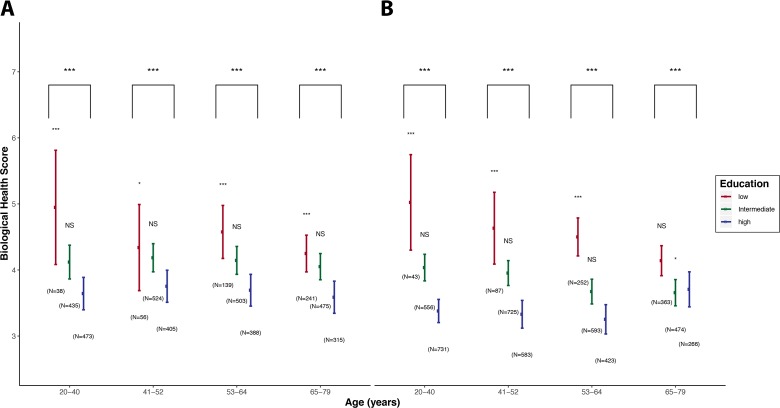
BHS distributions by age groups and educational levels. For each category the point estimate of the mean BHS is represented by a bullet, and the vertical line represents the 2.5%–97.5% CI of the score in that category. Low, intermediate and high education are represented in red, green and blue, respectively, and results are presented for men (A) and women (B) separately. For both genders and within each age class, potential differences in mean BHS by SEP (Socio Economic Position) category are tested using a Student’s t-test, setting the mean BHS in the high education category as a reference. We report the corresponding p value above the boxplots for low and intermediate education groups. Trends across the three education categories for both genders and within each age class were tested for using a Kruskal-Wallis test, and the corresponding p value is reported in the upper part of the plots. For readability, p values were coded as * for p values in (0.05, 0.01), ** for p values in (0.01, 0.001) and *** for p values <0.001. BHS, Biological Health Score.

BHS values calculated for each age and educational group in men (figure 1A) and women (figure 1B) show differences across age groups (p<0.05). Irrespective of age and gender we found a clear and consistent social gradient in BHS leading to higher scores (ie, higher biological risk) in participants with lower education.

Similarly, in both genders we observed values of BHS in the intermediate SEP category which were higher (p<0.05) than those observed in the high education group, for all age groups, except for women aged 65–79 years.

Similar analyses were conducted on each system-specific score (table 2). In both genders and in all age groups, we found statistically significant trends (p<0.001, not shown) for all systems. We found higher system-specific subscores in the low education group, for all systems except for kidney function. The comparison of the mean system-specific scores within each age group showed that the differences we identified were consistent in the low-to-high education group comparisons.

**Table 2:** Mean (2.5–97.5% CI of the mean) of the standardised system-specific subscore

Age	20–40 years old			41–52 years old			53–64 years old			65–79 years old		
Education	Low	Intermediate	High	Low	Intermediate	High	Low	Intermediate	High	Low	Intermediate	High
**(A) Men**												
Endocrine system	0.30 (0.20 to 0.40) (0.222)	0.25 (0.22 to 0.28) (0.570)	0.24 (0.21 to 0.27)	0.26 (0.18 to 0.34) (0.514)	0.26 (0.23 to 0.29) (0.135)	0.23 (0.20 to 0.26)	0.28 (0.23 to 0.33) (0.158)	0.25 (0.22 to 0.28) (0.475)	0.23 (0.20 to 0.27)	**0.27** **(0.23** **to 0.31**) (**0.007**)	**0.27** **(0.24** **to 0.30**) (**0.002**)	0.20 (0.17 to 0.23)
Metabolic system	**0.40** **(0.32** **to 0.48**) (**1.42e-04**)	**0.28** **(0.25** **to 0.30**) (**0.009**)	0.23 (0.21 to 0.26)	0.28 (0.21 to 0.34) (0.467)	**0.29** **(0.27** **to 0.31**) (**0.009**)	0.25 (0.23 to 0.27)	**0.29** **(0.25** **to 0.33**) (**0.031**)	0.26 (0.24 to 0.28) (0.197)	0.24 (0.22 to 0.26)	**0.28** **(0.25** **to 0.31**) (**0.004**)	**0.26** **(0.24** **to 0.28**) (**0.017**)	0.22 (0.19 to 0.25)
Cardiovascular system	0.21 (0.11 to 0.31) (0.901)	0.20 (0.17 to 0.23) (0.949)	0.20 (0.18 to 0.23)	0.20 (0.12 to 0.27) (0.918)	0.21 (0.19 to 0.24) (0.600)	0.20 (0.17 to 0.23)	0.19 (0.14 to 0.24) (0.683)	0.22 (0.20 to 0.25) (0.417)	0.21 (0.18 to 0.24)	0.23 (0.20 to 0.27) (0.830)	0.22 (0.19 to 0.24) (0.306)	0.24 (0.21 to 0.27)
Inflammatory system	**0.35** **(0.26** **to 0.45**) (**0.021**)	**0.28** **(0.25** **to 0.31**) (**0.026**)	0.23 (0.21 to 0.26)	**0.38** **(0.3** **to 0.46**) (**0.002**)	**0.28** **(0.26** **to 0.31**) (**0.004**)	0.23 (0.20 to 0.25)	**0.41** **(0.37** **to 0.46**) (**1.96e-10**)	**0.28** **(0.25** **to 0.30**) (**0.018**)	0.23 (0.20 to 0.26)	**0.30** **(0.27** **to 0.34**) (**1.46e-04**)	**0.28** **(0.25** **to 0.30**) (**9.04e-04**)	0.21 (0.18 to 0.24)
Liver system	0.31 (0.2 to 0.41) (0.091)	**0.27** **(0.24** **to 0.30**) (**0.012**)	0.21 (0.18 to 0.24)	0.27 (0.19 to 0.36) (0.570)	0.26 (0.23 to 0.28) (0.707)	0.25 (0.21 to 0.28)	0.28 (0.22 to 0.33) (0.143)	0.27 (0.24 to 0.29) (0.132)	0.23 (0.20 to 0.26)	0.23 (0.19 to 0.27) (0.589)	0.24 (0.21 to 0.27) (0.844)	0.25 (0.21 to 0.28)
Kidney system	**0.13** **(−0.01** **to 0.27**) (**0.019**)	0.26 (0.21 to 0.3) (0.503)	0.27 (0.24 to 0.31)	0.16 (0.05 to 0.27) (0.088)	0.25 (0.21 to 0.28) (0.478)	0.27 (0.22 to 0.31)	0.18 (0.11 to 0.25) (0.117)	0.3 (0.26 to 0.33) (0.088)	0.24 (0.2 to 0.29)	0.28 (0.22 to 0.33) (0.091)	0.26 (0.22 to 0.3) (0.167)	0.22 (0.17 to 0.26)

(B) Women												
	20–40 years old			41–52 years old			53–64 years old			65–79 years old		
	Low	Intermediate	High	Low	Intermediate	High	Low	Intermediate	High	Low	Intermediate	High
Endocrine system	0.32 (0.18 to 0.45) (0.592)	**0.22** **(0.18** **to 0.26**) (**0.015**)	0.28 (0.25 to 0.31)	0.28 (0.19 to 0.38) (0.944)	0.25 (0.21 to 0.28) (0.189)	0.28 (0.24 to 0.31)	**0.36** **(0.31** **to 0.42**) (**3.88e-04**)	0.28 (0.24 to 0.31) (0.086)	0.23 (0.19 to 0.27)	0.29 (0.25 to 0.34) (0.500)	0.27 (0.23 to 0.31) (0.981)	0.27 (0.21 to 0.32)
Metabolic system	**0.36** **(0.28** **to 0.44**) (**0.001**)	**0.30** **(0.28** **to 0.32**) (**8.39e-06**)	0.23 (0.22 to 0.25)	**0.35** **(0.29** **to 0.41**) (**4.84e-05**)	**0.26** **(0.25** **to 0.28**) (**0.008**)	0.23 (0.20 to 0.25)	**0.31** **(0.28** **to 0.34**) (**1.02e-04**)	0.25 (0.23 to 0.27) (0.139)	0.22 (0.20 to 0.25)	**0.32** **(0.29** **to 0.34**) (**3.94e-04**)	0.25 (0.23 to 0.27) (0.610)	0.24 (0.21 to 0.27)
Cardiovascular system	0.23 (0.14 to 0.32) (0.238)	**0.24** **(0.21** **to 0.26**) (**6.14e-04**)	0.18 (0.16 to 0.20)	0.23 (0.17 to 0.30) (0.304)	0.21 (0.19 to 0.23) (0.410)	0.20 (0.17 to 0.22)	0.22 (0.19 to 0.26) (0.697)	0.21 (0.18 to 0.23) (0.708)	0.21 (0.19 to 0.24)	0.22 (0.19 to 0.25) (0.406)	0.21 (0.19 to 0.24) (0.272)	0.24 (0.20 to 0.27)
Inflammatory system	**0.39** **(0.30** **to 0.48**) (**5.09e-04**)	**0.28** **(0.26** **to 0.31**) (**0.001**)	0.23 (0.20 to 0.25)	**0.41** **(0.35** **to 0.47**) (**1.33e-09**)	**0.31** **(0.29** **to 0.33**) (**9.76e-10**)	0.21 (0.19 to 0.23)	**0.35** **(0.31** **to 0.39**) (**9.09e-09**)	**0.26** **(0.24** **to 0.29**) (**0.002**)	0.20 (0.18 to 0.23)	**0.29** **(0.26** **to 0.32**) (**0.045**)	0.26 (0.23 to 0.28) (0.393)	0.24 (0.21 to 0.27)
Liver system	**0.43** **(0.34** **to 0.53**) (**7.93e-05**)	**0.27** **(0.25** **to 0.30**) (**0.040**)	0.24 (0.21 to 0.26)	0.27 (0.20 to 0.34) (0.172)	**0.28** **(0.26** **to 0.30**) (**0.002**)	0.22 (0.20 to 0.25)	**0.30** **(0.26** **to 0.34**) (**4.89e-04**)	**0.25** **(0.23** **to 0.28**) (**0.029**)	0.21 (0.18 to 0.24)	0.26 (0.23 to 0.29) (0.751)	0.25 (0.22 to 0.28) (0.403)	0.27 (0.23 to 0.31)
Kidney system	**0.12** **(−0.01** **to 0.25**) (**0.012**)	0.25 (0.22 to 0.29) (0.997)	0.25 (0.22 to 0.28)	0.21 (0.12 to 0.30) (0.255)	0.24 (0.21 to 0.28) (0.408)	0.26 (0.23 to 0.30)	0.30 (0.24 to 0.35) (0.123)	0.24 (0.21 to 0.28) (0.970)	0.24 (0.20 to 0.29)	0.30 (0.26 to 0.35) (0.084)	0.24 (0.20 to 0.28) (0.895)	0.24 (0.19 to 0.29)

Results presented in men (A) and women (B) separately. Differences in mean system-specific subscores were tested using a Student’s t-test setting for each age group and gender separately, setting the mean score observed for the high education group in that age group and gender as a reference. The corresponding p value is reported in parentheses. Scores showing statistically different means are represented in bold.

The strongest differences were observed for the inflammatory subscore, which showed (for all age groups in both men and women) higher mean values in participants with lower education. The metabolic subscore was also found to be statistically higher in the low education group in women (for all age groups; table 2B) and in men (for all age groups except for the 41–52 years group; table 2A). Differences in the mean metabolic subscore in the intermediate education group were weaker and less consistent across age groups.

**Figure 2:**
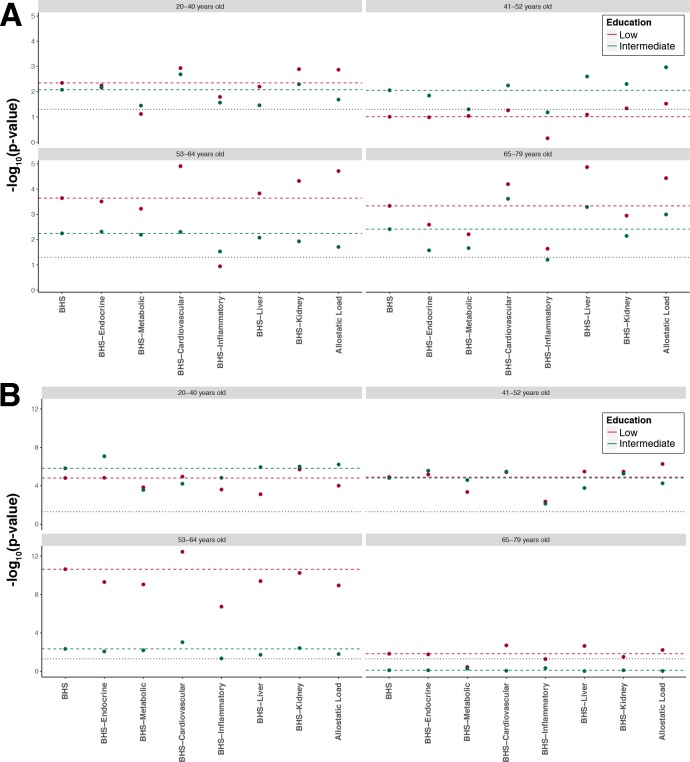
Sensitivity analysis investigating changes in p value induced by the exclusion of each system separately from the Biological Health Score (BHS). We report on the x axis the different scores considered (ie, removing one system at a time or both kidney and liver functions to mimic the allostatic load). The p value reported on the y axis measures, in each age group and gender, the significance of the difference between the mean score in the low (red) and intermediate (green) educational level group compared with the mean score in the reference group (higher education). As a reference, we report results from the full BHS (horizontal coloured dashed line). Results are presented for each age class separately in men (A) and women (B), and the black horizontal dotted lines represent the 0.05 significance level.

Excluding from the BHS (1) one biological system at a time and (2) both liver and kidney functions, we found that both in men (figure 2A) and women (figure 2B), and across all four age groups, the exclusion of the inflammatory system attenuates the differences in the mean score especially in men aged 41–52 years and 53–64 years, where differences lose statistical significance (p>0.05). Conversely, the exclusion of the cardiovascular system seems to strengthen the differences in all age groups in men and women.

**Figure 3:**
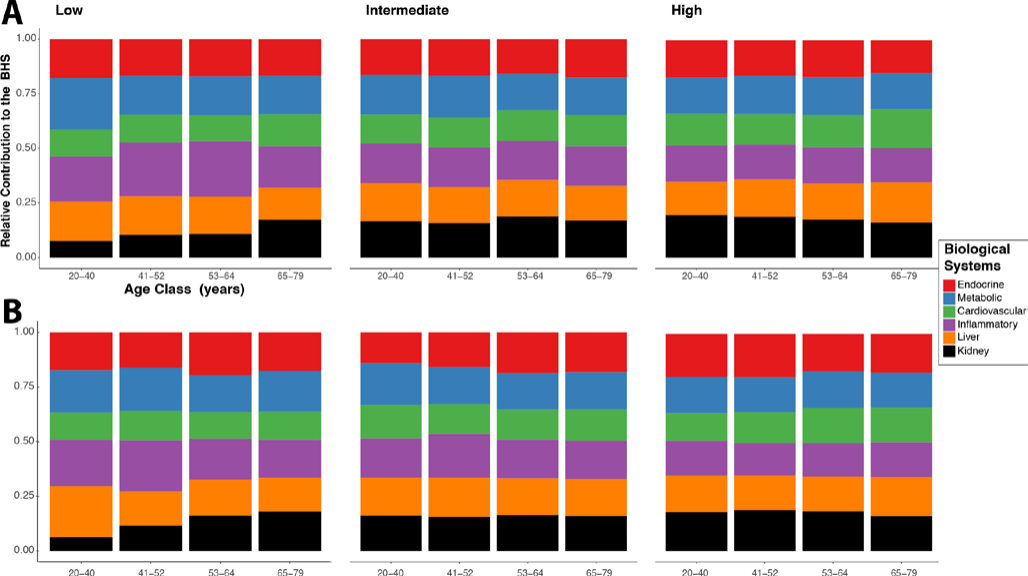
Relative contribution of each system-specific subscore to the Biological Health Score (BHS). Results are presented for men (A) and women (B) separately, and for each group of educational level: low (left), intermediate (middle) and high (right). To account for the differential number of biomarkers assayed in each system, the contribution is calculated based on a normalised system-specific score.

The relative contribution of each system to the BHS in men (figure 3A) and women (figure 3B) shows all system contributed to the BHS. However, their relative contribution varies across age groups in participants with lower education. These differential contributions are attenuated in the intermediate education group and almost non-existent in the higher education group.

The linear regression coefficients reported in table 3 represent the average adjusted contribution of education to the BHS. Results from the unadjusted model (model 1) suggest that BHS in the low and intermediate education groups are higher than in the high education group (p<0.05) in both men (table 3A) and women (table 3B) across all age groups, except for men in the low education group in the 20–40 years and 41–52 years age groups and in women with intermediate education group aged 65–79 years old. Differences between low and high education groups (p<0.05) are mostly observed in men older than 53 years and in women from the first three age groups. In women from the 20–40 years and 53–64 years age groups and in men from the 53–64 years age group, the average differences in BHS between low and high education groups are attenuated on adjustment for comorbidity, treatment, behaviours and BMI, but remained below 0.05 throughout the six different models. For the other age groups, the observed differences in BHS were attenuated (p>0.05) on adjustment for behaviours (model 6) in men and women (model 5). This suggests that the association between BHS values in low and high education groups cannot be fully explained by the covariates in these age groups.

**Table 3:** Mean (and SE) contribution of education to the BHS by age group

(A) Men	Model 1	Model 2	Model 3	Model 4	Model 5	Model 6
	β (SE) p value	β (SE) p value	β (SE) p value	β (SE) p value	β (SE) p value	β (SE) p value
20–40 years old (reference high SEP, n=381)						
Low (n=29)	0.84 (0.53) 1.09e-01	0.86 (0.52) 1.01e-01	0.76 (0.52) 1.40e-01	0.56 (0.52) 2.79e-01	0.59 (0.52) 2.63e-01	0.29 (0.46) 5.24e-01
Intermediate (n=331)	**0.4** (0.2) **5.12e-02**	**0.42** (0.2) **3.80e-02**	**0.43** (0.2) **3.52e-02**	0.36 (0.2) 7.70e-02	0.36 (0.2) 8.06e-02	0.01 (0.18) 9.47e-01
41–52 years old (reference high SEP, n=339)						
Low (n=39)	0.42 (0.42) 3.17e-01	0.45 (0.43) 2.92e-01	0.4 (0.42) 3.40e-01	0.4 (0.42) 3.44e-01	0.09 (0.42) 8.39e-01	0.04 (0.39) 9.28e-01
Intermediate (n=441)	**0.43** (0.18) **1.70e-02**	**0.43** (0.18) **1.76e-02**	**0.44** (0.18) **1.39e-02**	**0.45** (0.18) **1.18e-02**	**0.37** (0.18) **4.14e-02**	0.19 (0.17) 2.61e-01
53–64 years old (reference high SEP, n=330)						
Low (n=108)	**0.86** (0.27) **1.30e-03**	**0.77** (0.27) **4.11e-03**	**0.69** (0.26) **8.97e-03**	**0.65** (0.26) **1.45e-02**	**0.67** (0.28) **1.51e-02**	**0.55** (0.26) **3.60e-02**
Intermediate (n=436)	**0.39** (0.17) **2.68e-02**	**0.37** (0.17) **3.54e-02**	0.32 (0.17) 6.58e-02	0.31 (0.17) 6.84e-02	0.3 (0.18) 8.53e-02	0.2 (0.17) 2.30e-01
65–79 years old (reference high SEP, n=277)						
Low (n=207)	**0.58** (0.2) **4.51e-03**	**0.54** (0.2) **7.70e-03**	**0.51** (0.2) **1.21e-02**	**0.45** (0.2) **2.66e-02**	0.33 (0.21) 1.11e-01	0.25 (0.2) 2.13e-01
Intermediate (n=407)	**0.44** (0.17) **1.12e-02**	**0.38** (0.17) **2.86e-02**	**0.35** (0.17) **4.34e-02**	0.33 (0.17) 5.44e-02	0.26 (0.17) 1.35e-01	0.18 (0.16) 2.63e-01

(B) Women	Model 1	Model 2	Model 3	Model 4	Model 5	Model 6
	β (SE) p value	β (SE) p value	β (SE) p value	β (SE) p value	β (SE) p value	β (SE) p value
20–40 years old (reference high SEP, n=581)						
Low (n=28)	**2.1** (0.47) **1.01e-05**	**2.07** (0.47) **1.45e-05**	**1.95** (0.46) **2.54e-05**	**1.73** (0.46) **1.61e-04**	**1.66** (0.46) **2.84e-04**	**1.02** (0.41) **1.22e-02**
Intermediate (n=445)	**0.63** (0.15) **4.59e-05**	**0.63** (0.15) **5.46e-05**	**0.54** (0.15) **4.00e-04**	**0.56** (0.15) **1.90e-04**	**0.54** (0.15) **3.24e-04**	**0.33** (0.13) **1.53e-02**
41–52 years old (reference high SEP, n=508)						
Low (n=68)	**1.23** (0.34) **2.52e-04**	**1.21** (0.34) **3.23e-04**	**0.87** (0.32) **7.41e-03**	**0.74** (0.32) **2.14e-02**	0.33 (0.33) 3.15e-01	0.2 (0.3) 5.01e-01
Intermediate (n=622)	**0.61** (0.16) **9.20e-05**	**0.59** (0.16) **1.66e-04**	**0.49** (0.15) **1.17e-03**	**0.48** (0.15) **1.19e-03**	**0.31** (0.15) **3.50e-02**	0.19 (0.13) 1.50e-01
53–64 years old (reference high SEP, n=367)						
Low (n=213)	**1.27** (0.2) **1.91e-10**	**1.24** (0.2) **6.67e-10**	**1.01** (0.2) **4.52e-07**	**0.95** (0.2) **2.08e-06**	**0.73** (0.2) **3.63e-04**	**0.58** (0.19) **2.57e-03**
Intermediate (n=520)	**0.37** (0.16) **1.69e-02**	**0.38** (0.16) **1.66e-02**	0.3 (0.15) 5.13e-02	0.3 (0.15) 5.53e-02	0.2 (0.16) 2.07e-01	0.09 (0.15) 5.42e-01
65–79 years old (reference high SEP, n=279)						
Low (n=294)	**0.47** (0.19) **1.45e-02**	**0.45** (0.19) **2.11e-02**	0.35 (0.19) 7.28e-02	0.32 (0.19) 1.01e-01	0.27 (0.2) 1.82e-01	0.17 (0.19) 3.76e-01
Intermediate (n=409)	−0.08 (0.18) 6.50e-01	−0.1 (0.18) 5.95e-01	−0.14 (0.18) 4.36e-01	−0.16 (0.18) 3.85e-01	−0.19 (0.18) 2.93e-01	−0.19 (0.17) 2.75e-01

Results are presented in men (A) and women (B) separately and effects are calculated for the unadjusted model (model 1) and for models sequentially adjusting for marital status and overcrowding (model 2), comorbidities (model 3), medical treatments (model 4), smoking, physical activity and alcohol (model 5), and BMI (model 6). These analyses are restricted to (n=3325 men and n=4334 women) participants with full information on the confounders included in these six models. For each age group, the difference between the effect estimated in the low and intermediate SEP (Socio Economic Position) groups and that of the high SEP group was tested using a Student’s t-test, and differences with p values below the nominal 0.05 significance level are reported in bold.

BHS, Biological Health Score; BMI, body mass index.

We found differences (p<0.05) in mean BHS between intermediate and high education groups, in both gender for all age groups, except for women aged 65–79 years. These differences appeared independent of all covariates considered in women from the 20–40 age group.

## Discussion

### Main findings

In this study, we extended the concept of the AL in a multisystem BHS measuring features of the four physiological systems originally included in the AL (endocrine, inflammatory, cardiovascular and metabolic systems) and the function of two key organs (liver and kidney). We found a persistent socioeconomic gradient in the BHS in men and women and across age groups, leading to the more socially disadvantaged being at greater biological risk. Because the score was calculated using the distribution of the biomarkers in each gender and age group separately (see box 1), our estimates of the BHS do not capture the natural age-related changes in the individual level of functioning. As such, the slight changes in BHS we observe across age groups cannot be interpreted as being related to the natural age-related variability of the BHS. We found that each system contributed to the BHS and its differential distribution by age, gender and SEP categories, but we identified that the inflammatory system and, to a lesser extent, the metabolic system consistently drove (across age groups and gender) the observed values of the BHS. Overall our analyses suggest that the effect of SEP is detected for most biological systems, independently of the SEP-related covariates and is generally more consistent in women.

SEP was associated with biological health risk from early adulthood, and this association was not explained by covariates such as comorbidity or lifestyle in this sample of UK residents. These findings highlight evidence of social-to-biological processes leading to health inequalities from early adulthood.

### Comparison with previous studies

Overall, our findings are consistent with work carried out in other dominantly white populations from high-income countries using the multibiomarker indicator AL. This confirms that this type of aggregate score, which summarises information across different biological systems, is able to capture a dimension of physiological health above and beyond individual biomarkers or systems.^9 21^ Our sensitivity analyses reinforce this message, showing that adding the extra systems (here limited to liver and kidney) to the AL indicator may pick up additional differences in physiological function or dysfunction leading to pathologies. As such our study warrants future and further extensions of the AL so that it includes additional systems that may contribute to social gradients in health.

In order to ascertain possible modifications to the pattern of biological risk across age groups, we sequentially linked education and BHS adjusting for possible confounders. In several models, the relationship between education and biological health was attenuated after adjusting for comorbidities, treatments and behaviours. These findings are consistent with the increase in comorbidities associated with lower SEP and increased age, and consequently in treatments for diseases or biomarkers that may contribute to our BHS. Our results are also consistent with the well-established association linking SEP and behaviours and obesity. BMI may thus appear as a particularly important mediator of the association between education and biological functioning/risk in this population. It is however noteworthy that the association between lower education and higher BHS remained statistically significant after adjustment for all covariates in some age groups (53–64 years in men, and 20–40 years and 53–64 years in women), hence suggesting that other mechanisms are involved in the way education exerts its effect on biological functioning and health.

### Strengths and weaknesses of the study

The large population size from a wide age range and the broad panel of biomarkers available, allowing us to adopt a systems approach, represent a key strength of this study. Most other studies focusing on biological wear-and-tear and biological health are in middle-aged or elderly participants, especially when multiple biomarkers are used. Here, we were able to examine whether socially patterned differences in biological health were observed in young adults. Nevertheless, to perform our analyses, and in line with previous investigations using *Understanding Society*, a large fraction (n=25 700 out of 35 875) of the study participants were excluded based on the availability of both (1) a blood sample and (2) a full nurse assessment. As a result, the included population is not representative of the general UK population anymore.^15^ Individual weights capturing differential response rates across population subgroups have been developed and could be applied to each observation in our study population to ensure the applicability of our results to the general population. However, these would result in a substantial drop in the number of effective observations in the younger age groups, which represents a particularly important subpopulation in our study. For that reason, we decided to restrict our work to unweighted analyses and carefully interpreted our findings, avoiding any generalisation to the full UK population.

The main limitation of our work resides in its cross-sectional nature. By construction we cannot discern if the social differences in biological health observed in the younger age group persist across the life course, or whether these relationships are specific to the age, calendar time and demographic structure of this cross-sectional population. In particular, our study population does not allow to quantify to which extent the age-related differences in BHS can be attributed to age itself or to calendar time differentials in the exposures and experiences captured by education. This calls for a cautious interpretation of our findings; to disentangle the age and calendar contribution to the effects we detect, analysis of longitudinal data is warranted. In addition, due to its cross-sectional nature, comorbidities reported in our study are prevalent. Therefore, our conclusion that the BHS may be able to capture existing pathological processes should not be misinterpreted in a causal or predictive context.

We used a straightforward method relying on dichotomising the level of each biomarker to calculate the BHS, which has been used to construct the AL score in previous works. The construction of such synthetic scores relying on the discretisation of the biomarkers level, and its present application defining the biomarkers quantiles for each gender and age group separately, makes our inference robust to the way each system is measured. For instance, glomerular filtration rate (GFR) could be used instead of creatinine levels to measure kidney function. However, by construction the ranking of the participants (within each age group and sex), according to either the GFR and creatinine levels, will remain unchanged. Hence, the creatinine to GFR transformation would not have any impact on our subsequent inferences. Other methods can be employed to formulate a measure of biological health and have been used to examine the internal consistency and dimensionality of these types of multibiomarker scores.^22 23^ Overall this relatively simple approach, which uses the sample distribution of each biomarker to define the group most ‘at risk’, ultimately creating a single synthetic score, has been shown to successfully capture a common variance^21^ between AL components as hypothesised by the originators.^1 9^ This type of targeted approaches relying on established and functionally characterised biomarkers could be complemented by more agnostic investigations as exemplified by the recent development of biological clock using high-throughput data, and in particular DNA methylation.^24–26^ The clocks have been shown to be related to ageing, mortality, and more recently to be affected by social adversity.^27^ As such, one natural extension of our work would be to screen for molecular signatures of our BHS extension of the AL and to evaluate how these are related to the CpG sites contributing to the established methylation clocks.

## Conclusions and implication for future research

Our findings highlight that social-to-biological processes leading to health inequalities adversely affect biological health from early adulthood and independently from social-related exposures. We show that social differences in biological risk cannot be fully^24–26^ explained by lifestyle or behavioural factors, which warrants further investigations to identify the additional ageing-relevant information that the kind of biological risk measure brings about.

Disadvantaged social conditions should be considered as a precursor to accelerated biological ageing from early adulthood and as serious targets for public health policies. Social-to-biological processes beginning in the childhood environment are likely to form a chain of events that lead to socially differentiated biological states and health inequalities. Such processes could be at play in the recent shifts in life expectancy observed in high-income countries.^28^ Policy makers working on child care provision, housing and educational facilities must consider these areas as risk factors for social-to-biological processes involved in the construction of health inequalities.

**What is already known on this subject**

- It is established that social position impacts the quality of ageing, through the stimulation/dysregulation of key physiological systems during the life course.
- Composite scores as the allostatic load can be used to measure individual physiological wear-and-tear, and scores focusing on the physiological response to stress have shown social gradients in biological risk among the elderly.

**What this study adds**

- In the present study, as a potential extension of the allostatic load, we include two additional key systems and organs not primarily involved in the stress response, and analyse this extended score in a large age range from early to late adulthood.
- We show that education-related physiological differentials can already be detected in early adulthood (20–40 years age group) and that these are not solely driven by lifestyle and behavioural factors.
- We show that while both the inflammatory and metabolic systems have a consistent contribution to biological risk throughout the life course, other systems seem to differentially impact the Biological Health Score at different ages.
- Our findings suggest that the social-to-biological processes leading to health inequalities can be detected early in life and therefore highlight early adulthood disadvantaged social condition as a precursor to accelerated biological ageing.
