# Supplementary material for: Early-life inequalities and biological ageing: a multisystem Biological Health Score approach in U nderstanding S ociety
Source: J Epidemiol Community Health. 2019 Apr 3;73(8):693–702. doi: 10.1136/jech-2018-212010 (PMC6678052; doi:10.1136/jech-2018-212010)
Supplement: Supplementary data [file jech-2018-212010supp001.pdf]

Supplementary Table 1. Description of the study population across all covariates by gender and age class. The percentages given in parenthesis are calculated as the proportion of the number of individuals in a covariate's level of an age class and gender to the total number of individuals in that age class and gender.

	Women (%)				Men (%)			
	20-40 (N=1330)	41-52 (N=1395)	53-64 (N=1268)	65-79 (N=1103)	20-40 (N=946)	41-52 (N=985)	53-64 (N=1030)	65-79 (N=1031)
**Education**								
Low	43 (3.23)	87 (6.24)	252 (19.87)	363 (32.91)	38 (4.02)	56 (5.69)	139 (13.5)	241 (23.38)
Intermediate	556 (41.8)	725 (51.97)	593 (46.77)	474 (42.97)	435 (45.98)	524 (53.2)	503 (48.83)	475 (46.07)
High	731 (54.96)	583 (41.79)	423 (33.36)	266 (24.12)	473 (50)	405 (41.12)	388 (37.67)	315 (30.55)
**Parental Education**								
Low	116 (8.72)	275 (19.71)	431 (33.99)	431 (39.07)	76 (8.04)	173 (17.56)	348 (33.79)	422 (40.93)
Intermediate	286 (21.50)	266 (19.07)	153 (12.07)	120 (10.88)	201 (21.25)	213 (21.62)	154 (14.95)	99 (9.60)
High	493 (37.07)	415 (29.75)	282 (22.24)	207 (18.77)	366 (38.69)	286 (29.04)	208 (20.19)	174 (16.88)
Missing	435 (32.71)	439 (31.47)	402 (31.70)	345 (31.28)	303 (32.03)	313 (31.78)	320 (31.07)	336 (32.59)
**Marital status**								
Single	368 (27.67)	125 (8.96)	55 (4.34)	52 (4.71)	267 (28.22)	111 (11.27)	90 (8.74)	51 (4.95)
Living as couple or married	886 (66.62)	1014 (72.69)	913 (72)	654 (59.29)	662 (69.98)	778 (78.98)	786 (76.31)	802 (77.79)
Separated or divorced	72 (5.41)	240 (17.2)	228 (17.98)	121 (10.97)	17 (1.8)	92 (9.34)	119 (11.55)	101 (9.8)
Widowed	4 (0.3)	16 (1.15)	72 (5.68)	276 (25.02)	0 (0)	4 (0.41)	35 (3.4)	77 (7.47)
**Household overcrowding**								
No	1083 (81.43)	1222 (87.6)	1173 (92.51)	1035 (93.83)	753 (79.6)	846 (85.89)	959 (93.11)	958 (92.92)
Yes	246 (18.5)	173 (12.4)	95 (7.49)	68 (6.17)	192 (20.3)	138 (14.01)	70 (6.8)	73 (7.08)
Missing	1 (0.08)	0 (0)	0 (0)	0 (0)	1 (0.11)	1 (0.1)	1 (0.1)	0 (0)
**Co-morbidities**								
None	784 (58.95)	714 (51.18)	411 (32.41)	225 (20.4)	600 (63.42)	543 (55.13)	381 (36.99)	233 (22.6)
One	354 (26.62)	383 (27.46)	414 (32.65)	381 (34.54)	218 (23.04)	268 (27.21)	336 (32.62)	324 (31.43)
Two or more	111 (8.35)	256 (18.35)	408 (32.18)	475 (43.06)	43 (4.55)	119 (12.08)	266 (25.83)	448 (43.45)
Missing	81 (6.09)	42 (3.01)	35 (2.76)	22 (1.99)	85 (8.99)	55 (5.58)	47 (4.56)	26 (2.52)
**Number of treatments**								
None	710 (53.38)	662 (47.46)	415 (32.73)	183 (16.59)	756 (79.92)	626 (63.55)	417 (40.49)	177 (17.17)
One	420 (31.58)	381 (27.31)	350 (27.6)	302 (27.38)	145 (15.33)	212 (21.52)	290 (28.16)	269 (26.09)
Two or more	200 (15.04)	352 (25.23)	503 (39.67)	618 (56.03)	45 (4.76)	147 (14.92)	323 (31.36)	585 (56.74)
**Smoking**								
Never	546 (41.05)	640 (45.88)	556 (43.85)	503 (45.6)	381 (40.27)	405 (41.12)	318 (30.87)	265 (25.7)
Yes	784 (58.95)	755 (54.12)	712 (56.15)	600 (54.4)	565 (59.73)	580 (58.88)	712 (69.13)	766 (74.3)
**Sports activity**								
At least one sport	984 (73.98)	929 (66.59)	761 (60.02)	610 (55.3)	734 (77.59)	722 (73.3)	671 (65.15)	622 (60.33)
None	346 (26.02)	466 (33.41)	507 (39.98)	493 (44.7)	212 (22.41)	263 (26.7)	359 (34.85)	409 (39.67)
**Alcohol consumption**								
Non-drinker	146 (10.98)	166 (11.9)	166 (13.09)	168 (15.23)	54 (5.71)	48 (4.87)	76 (7.38)	86 (8.34)
Social drinker	354 (26.62)	352 (25.23)	289 (22.79)	219 (19.85)	275 (29.07)	306 (31.07)	279 (27.09)	266 (25.8)
Moderate drinker	433 (32.56)	364 (26.09)	337 (26.58)	335 (30.37)	236 (24.95)	195 (19.8)	160 (15.53)	193 (18.72)
Daily drinker	206 (15.49)	374 (26.81)	364 (28.71)	276 (25.02)	267 (28.22)	332 (33.71)	430 (41.75)	409 (39.67)
Missing	191 (14.36)	139 (9.96)	112 (8.83)	105 (9.52)	114 (12.05)	104 (10.56)	85 (8.25)	77 (7.47)
**Body Mass index** (Kg/m^2)								
Under 18.5	30 (2.26)	11 (0.79)	9 (0.71)	10 (0.91)	10 (1.06)	3 (0.3)	4 (0.39)	4 (0.39)
18.5 and below 25	548 (41.2)	475 (34.05)	340 (26.81)	289 (26.2)	314 (33.19)	193 (19.59)	221 (21.46)	193 (18.72)
25 and below 30	392 (29.47)	448 (32.11)	463 (36.51)	396 (35.9)	411 (43.45)	439 (44.57)	467 (45.34)	485 (47.04)
30 and below 40	288 (21.65)	366 (26.24)	366 (28.86)	333 (30.19)	184 (19.45)	314 (31.88)	291 (28.25)	286 (27.74)
above 40	54 (4.06)	70 (5.02)	58 (4.57)	25 (2.27)	14 (1.48)	23 (2.34)	19 (1.84)	19 (1.84)
Missing	18 (1.35)	25 (1.79)	32 (2.52)	50 (4.53)	13 (1.37)	13 (1.32)	28 (2.72)	44 (4.27)
