# Supplementary material for: Early-life inequalities and biological ageing: a multisystem Biological Health Score approach in U nderstanding S ociety
Source: J Epidemiol Community Health. 2019 Apr 3;73(8):693–702. doi: 10.1136/jech-2018-212010 (PMC6678052; doi:10.1136/jech-2018-212010)
Supplement: Supplementary data [file jech-2018-212010supp002.pdf]

Supplementary Table 2. Summary statistics of the biomarkers used to calculate the BHS. These include six physiological systems and results are presented for men (A) and women (B), and for each age group separately.

A Men

	Age class															
	20-40 years old				41-52 years old				53-64 years old				65-79 years old			
Biomarkers	1st Qu.	Mean	3rd Qu.	Missing (%)	1st Qu.	Mean	3rd Qu.	Missing (%)	1st Qu.	Mean	3rd Qu.	Missing (%)	1st Qu.	Mean	3rd Qu.	Missing (%)
Endocrine System																
Dehydroepiandrosterone (DHEA-S) (µmol/L)	6.7	8.92	10.8	31 (3.28)	4.2	6.21	7.77	27 (2.74)	2.8	4.45	5.7	22 (2.14)	1.7	3.02	3.9	39 (3.78)
Testosterone (nmol/L)	11.5	16.14	19.9	31 (3.28)	11	14.74	18.3	29 (2.94)	11.97	15.52	18.6	30 (2.91)	11.7	15.84	19.3	59 (5.72)
Metabolic System																
Glycosylated hemoglobin (HbA1c) (mmol/mol)	31	33.65	36	71 (7.51)	33	36.45	38	84 (8.53)	35	38.19	40	83 (8.06)	36	40.07	42	114 (11.06)
serum High-Density-Lipoprotein (HDL) (mmol/L)	1.1	1.36	1.6	26 (2.75)	1.1	1.35	1.6	30 (3.05)	1.1	1.4	1.6	30 (2.91)	1.1	1.4	1.6	43 (4.17)
Low-Density-Lipopretein (LDL) (mmol/L)	2.32	2.99	3.55	29 (3.07)	2.69	3.38	3.99	39 (3.96)	2.47	3.16	3.84	36 (3.5)	1.92	2.66	3.28	44 (4.27)
unfasted Triglycerides (TG) (mmol/L)	1.1	1.96	2.4	25 (2.64)	1.4	2.3	2.9	37 (3.76)	1.3	2.13	2.6	29 (2.82)	1.2	1.87	2.3	40 (3.88)
Cardio-vascular System																
Systolic blood pressure (SBP) (mmHg)	118.5	125.75	132.5	208 (21.99)	120.62	129.19	136	195 (19.8)	122.5	132.94	141.5	181 (17.57)	122.5	133.69	143.5	133 (12.9)
Diastolic blood pressure (DBP) (mmHg)	65	72.27	78.5	208 (21.99)	71	78.12	84	197 (20)	70	77.75	84	181 (17.57)	65	71.9	78	130 (12.61)
Pulses (beats/min)	59.5	67.27	74	210 (22.2)	60.5	68.4	75	195 (19.8)	60	67.67	74.5	182 (17.67)	58.5	66.19	72.5	130 (12.61)
Inflammatory/Immune System																
Fibrinogen (g/L)	2.1	2.45	2.8	26 (2.75)	2.3	2.67	3	34 (3.45)	2.4	2.79	3.1	20 (1.94)	2.6	2.92	3.2	40 (3.88)
C-reactive protein (CRP) (mg/L)	0.5	1.94	2	71 (7.51)	0.7	2.28	2.5	54 (5.48)	0.8	2.55	2.8	42 (4.08)	0.8	2.93	3.4	52 (5.04)
Insulin Growth Factor 1 (IGF-1) (nmol/L)	18	22.26	26	34 (3.59)	15	18.44	21	34 (3.45)	14	16.79	20	26 (2.52)	12	15.47	18	40 (3.88)
Liver Function																
Alanine transaminase (ALT) (U/L)	23	35.4	42.25	38 (4.02)	25	36.9	44	40 (4.06)	23	33.69	38	32 (3.11)	20	27.61	32	48 (4.66)
Aspartate transaminase (AST) (U/L)	27	33.1	37	69 (7.29)	28	33.59	37	77 (7.82)	27	32.9	36	76 (7.38)	26	31.45	35	84 (8.15)
Gamma glutamyltransferase (GGT) (U/L)	18	35.65	41	28 (2.96)	22	42.83	51	36 (3.65)	23	43.48	51	46 (4.47)	21	37.77	44	48 (4.66)
Kidney Function																
Creatinine (enzymatic) (µmol/L)	75	82.14	89	18 (1.9)	76	84.14	92	23 (2.34)	74	84.12	92	26 (2.52)	77	88.64	97	48 (4.66)

B Women

	Age class															
	20-40 years old				41-52 years old				53-64 years old				65-79 years old			
Biomarkers	1st Qu.	Mean	3rd Qu.	Missing (%)	1st Qu.	Mean	3rd Qu.	Missing (%)	1st Qu.	Mean	3rd Qu.	Missing (%)	1st Qu.	Mean	3rd Qu.	Missing (%)
Endocrine System																
Dehydroepiandrosterone (DHEA-S) (µmol/L)	3.5	5.49	7	60 (4.51)	2.5	4	5.2	36 (2.58)	1.6	2.78	3.6	36 (2.84)	1.1	2.09	2.7	34 (3.08)
Metabolic System																
Glycosylated hemoglobin (HbA1c) (mmol/mol)	31	33.37	35	117 (8.8)	32	35.01	37	120 (8.6)	35	37.73	40	115 (9.07)	36	39.21	41	124 (11.24)
serum High-Density-Lipoprotein (HDL) (mmol/L)	1.3	1.61	1.9	41 (3.08)	1.4	1.69	2	33 (2.37)	1.4	1.75	2	39 (3.08)	1.4	1.72	2	28 (2.54)
Low-Density-Lipopretein (LDL) (mmol/L)	2.16	2.7	3.16	47 (3.53)	2.5	3.1	3.64	39 (2.8)	2.75	3.4	4.03	52 (4.1)	2.41	3.18	3.92	33 (2.99)

unfasted Triglycerides (TG) (mmol/L)	0.8	1.29	1.6	45 (3.38)	0.9	1.51	1.9	38 (2.72)	1.1	1.73	2.2	48 (3.79)	1.2	1.75	2.1	30 (2.72)
Cardio-vascular System																
Systolic blood pressure (SBP) (mmHg)	106	113.14	119.5	270 (20.3)	109	119.17	127.5	262 (18.78)	115.75	127.43	137.5	213 (16.8)	121.62	133.21	143.88	149 (13.51)
Diastolic blood pressure (DBP) (mmHg)	63	69.96	76	270 (20.3)	67	74.13	81	262 (18.78)	68.5	75.05	82	214 (16.88)	65	71.6	78.25	148 (13.42)
Pulses (beats/min)	64	71	77.5	270 (20.3)	63	69.75	76	263 (18.85)	63	69.55	75.5	213 (16.8)	62.5	69.27	75.5	148 (13.42)
Inflammatory/Immune System																
Fibrinogen (g/L)	2.3	2.66	3	47 (3.53)	2.4	2.76	3	36 (2.58)	2.5	2.91	3.27	42 (3.31)	2.6	3	3.3	37 (3.35)
C-reactive protein (CRP) (mg/L)	0.6	2.93	3.3	87 (6.54)	0.6	2.62	2.9	77 (5.52)	0.8	3.24	3.8	70 (5.52)	0.9	3.34	3.88	53 (4.81)
Insulin Growth Factor 1 (IGF-1) (nmol/L)	18	22.73	27	58 (4.36)	14	17.86	21	41 (2.94)	12	15.13	18	40 (3.15)	11	14.24	17	30 (2.72)
Liver Function																
Alanine transaminase (ALT) (U/L)	15	21.79	25	56 (4.21)	16	22.77	26	59 (4.23)	19	25.55	30	56 (4.42)	18	23.92	27	35 (3.17)
Aspartate transaminase (AST) (U/L)	23	26.69	29	108 (8.12)	23	27.45	30	99 (7.1)	25	29.15	32	96 (7.57)	25	29.29	32	70 (6.35)
Gamma glutamyltransferase (GGT) (U/L)	12	20.61	23	49 (3.68)	13	24.37	28	51 (3.66)	16	29.54	34	58 (4.57)	16	27.86	32.75	41 (3.72)
Liver Function																
Creatinine (enzymatic) (µmol/L)	58	64.62	71	36 (2.71)	59	66.65	73	36 (2.58)	59	67.16	73	33 (2.6)	61	70.41	77	41 (3.72)
