# Supplementary material for: Early-life inequalities and biological ageing: a multisystem Biological Health Score approach in U nderstanding S ociety
Source: J Epidemiol Community Health. 2019 Apr 3;73(8):693–702. doi: 10.1136/jech-2018-212010 (PMC6678052; doi:10.1136/jech-2018-212010)
Supplement: Supplementary data [file jech-2018-212010supp003.pdf]

Supplementary Table 3. Distribution of the number of missing (and subsequently imputed) biomarkers. Results are presented for men and women separately. For each number of imputed variables, we report the cumulative proportion of the full study population with less than this number of imputed variables.

Number of Imputed Biomarkers	0	1	2	3	4	5	6	7	8	9	10	11	12	13	14	15
Men	2,782	524	98	608	149	28	9	2	9	7	2	22	28	39	6	7
Women	3,535	632	135	791	146	24	10	2	6	1	29	41	42	14	6	0
Total	6,317	1,156	233	1,399	295	52	19	4	15	8	31	63	70	53	12	7
Cumulative proportion of the population	64.9%	76.8%	79.2%	93.5%	96.6%	97.1%	97.3%	97.3%	97.5%	97.6%	97.9%	98.5%	99.3%	99.8%	99.9%	100%
